# Genome Sequence of the Human Abscess Isolate *Streptococcus intermedius* BA1

**DOI:** 10.1128/genomeA.00117-12

**Published:** 2013-01-31

**Authors:** Paul J. Planet, Ryan Rampersaud, Saul R. Hymes, Susan Whittier, Phyllis A. Della-Latta, Apurva Narechania, Sean C. Daugherty, Ivette Santana-Cruz, Robert DeSalle, Jacques Ravel, Adam J. Ratner

**Affiliations:** a Department of Pediatrics, Columbia University, New York, New York, USA; b Department of Pathology and Cell Biology, Columbia University, New York, New York, USA; c Clinical Microbiology Laboratory, NewYork-Presbyterian Hospital, New York, New York, USA; d Sackler Institute for Comparative Genomics, American Museum of Natural History, New York, New York, USA; e Institute for Genome Sciences, University of Maryland, School of Medicine, Baltimore, Maryland, USA

## Abstract

*Streptococcus intermedius* is a human pathogen with a propensity for abscess formation. We report a high-quality draft genome sequence of *S. intermedius* strain BA1, an isolate from a human epidural abscess. This sequence provides insight into the biology of *S. intermedius* and will aid investigations of pathogenicity.

## GENOME ANNOUNCEMENT

*Streptococcus intermedius* is a member of the anginosus or *Streptococcus milleri* group (SMG), which also includes *S. constellatus* and *S. anginosus* (1). This organism is part of the normal microbiota of human mucosal surfaces, including the upper respiratory and lower genital tracts, and can cause liver and brain abscesses, bacteremia, osteoarticular infections, and endocarditis (2–5). SMG bacteria are also newly recognized as likely pathogens in cystic fibrosis pulmonary infections (6). The strain BA1 was isolated from an intracranial abscess in a child who presented with complicated mastoiditis and osteomyelitis of the skull. The identification of the organism was confirmed by PCR and sequencing of the 16S rRNA genes and the gene encoding intermedilysin (ILY) (7).

The whole genome sequence of *S. intermedius* BA1 was determined using a hybrid approach including Roche/454 GS-FLX sequencing (593,795 reads; 378-bp average read length) and Pacific Biosciences (PacBio) R.S. sequencing (319,206 reads; 1,122-bp average read length). The sequencing errors of single-molecule reads were corrected by mapping 454 reads onto longer PacBio R.S. reads using an algorithm previously described (8). The resulting 24,599 corrected PacBio R.S. reads larger than 2,000 bp were assembled using Newbler (version 2.7) and yielded 49 contigs with a total size of 1,966,076 bp (*N*_50_, 209,077 bp) and 30-fold genome coverage. Fourteen long (>10-kbp) contigs contained 1,896,811 bp in total (96.5% of the genome sequence). Annotation was performed using the IGS prokaryotic annotation engine (9). The genome sequence has a G+C content of 37.8%, 63 tRNA genes, 6 rRNA genes, and 2,028 predicted coding sequences (with an average gene length of 848 nucleotides [nt]) comprising 87% of the genome.

The complement of putative virulence factors identified in the annotated draft genome sequence of *S. intermedius* BA1 has considerable similarity to those described for *S. pneumoniae. S. intermedius* BA1 contains a coding sequence for ILY, a human-specific pore-forming toxin closely related to vaginolysin from *Gardnerella vaginalis* and inerolysin from *Lactobacillus iners* (10, 11). ILY is important in *S. intermedius* pathogenesis (12–14), and its production is regulated by sensation of nutrient availability via the catabolite control protein A (15), which is also encoded in the *S. intermedius* BA1 draft genome sequence. Capsular polysaccharide synthesis (cps) is involved in immune evasion by pathogenic streptococci (16), and the *S. intermedius* BA1 genome encodes proteins predicted to be involved in cps pathways. Natural competence for transformation is a driving force in streptococcal evolution (17, 18), and competence pathway components similar to those of *S. pneumoniae* were identified in the BA1 genome, as was a predicted competence-stimulating peptide precursor. Genes for sortases and their predicted substrates containing consensus LPXTG motifs, two-component signaling systems, adhesins, sialidases, quorum-sensing components, and a eukaryotic-like serine-threonine protein kinase/phosphatase pair (PrkC/PrpC) were also identified. No predicted siderophore genes were noted in the BA1 genome.

### Nucleotide sequence accession numbers. 

The sequence from this Whole Genome Shotgun project has been deposited in DDBJ/EMBL/GenBank under accession number ANFT00000000 (BioProject PRJNA178554, SubID SUB138006). The version described in this paper is the first version, ANFT01000000.
